# Radiation dose reduction in chest dual-energy computed tomography: effect on image quality and diagnostic information

**DOI:** 10.1590/0100-3984.2017.0136

**Published:** 2018

**Authors:** Rodrigo Canellas, Subba Digumarthy, Azadeh Tabari, Alexi Otrakji, Shaunagh McDermott, Efren J. Flores, Mannudeep Kalra

**Affiliations:** 1 Department of Radiology, Division of Thoracic Imaging and Intervention, Massachusetts General Hospital, Boston, MA, USA.

**Keywords:** Radiation dose reduction, Dual energy computed tomography, Monochromatic images

## Abstract

**Objective:**

To determine whether dual-energy computed tomography (DECT) of the chest can be performed at a reduced radiation dose, with an emphasis on images generated with post-processing techniques.

**Materials and Methods:**

In 21 patients undergoing DECT of the chest in a dual-source scanner, an additional image series was acquired at a reduced radiation dose. Four thoracic radiologists assessed both image series for image quality, normal thoracic structures, as well as pulmonary and mediastinal abnormalities, on virtual monochromatic images at 40 keV and 60 keV. Data were analyzed with Student's t-test, kappa statistics, analysis of variance, and the Wilcoxon signed-rank test.

**Results:**

The overall image quality of 60 keV virtual monochromatic images at a reduced radiation dose was considered optimal in all patients, and no abnormalities were missed. Contrast enhancement and lesion detection performance were comparable between reduced-dose images at 40 keV and standard-of-care images at 60 keV. The intraobserver and interobserver agreement were both good. The mean volumetric CT dose index (CTDIvol), size-specific dose estimate (SSDE), dose-length product (DLP), and effective dose (ED) for reduced-dose DECT were 3.0 ± 0.6 mGy, 4.0 ± 0.6 mGy, 107 ± 30 mGy.cm, and 1.5 ± 0.4 mSv, respectively.

**Conclusion:**

DECT of the chest can be performed at a reduced radiation dose (CTDIvol < 3 mGy) without loss of diagnostic information.

## INTRODUCTION

Although the concept of dual-energy computed tomography (DECT) is almost as old as the CT technology itself, DECT initially required substantially higher radiation doses (nearly two times higher than that employed in single-energy CT) and presented problems associated with spatial misregistration of the two different kV image datasets between the two separate acquisitions^(^^1^^)^. In the 1990s, there was renewed interest in DECT for the characterization of solitary pulmonary nodules, several studies highlighting the value of DECT over single-energy CT techniques^(^^2^^)^. However, toward the end of that decade, a study sponsored by the Fleischner Society reported that DECT was not useful for pulmonary nodule characterization^(^^3^^)^.

Concerns over rising radiation doses from CT scanning have prompted several clinical studies and have led to the introduction of technologic advances aimed at reducing the radiation dose employed in CT^(^^4^^)^. Technological advances in multidetector CT have also enabled near simultaneous acquisition of DECT datasets, and some authors have reported that DECT can be performed at radiation doses similar to those employed in single-energy CT^(^^5^^)^. Subsequent studies of near-simultaneous DECT technologies reported several thoracic applications of DECT-such as the detection or evaluation of pulmonary embolisms, chronic pulmonary thromboembolic diseases, aortic aneurysm/dissection, and pulmonary nodules, as well as the differentiation between benign and malignant mediastinal lesions-in single-phase or dual-phase examinations^(^^6^^,^^7^^)^. Nevertheless, little attention has been given to the possibility of reducing the radiation dose received by patients undergoing DECT of the chest. Recent studies have reported that the post-processing of DECT images (the synthesis of virtual monochromatic images and the use of material separation techniques) is useful in the assessment of the lung parenchyma and of pulmonary embolisms^(^^8^^,^^9^^)^.

The purpose of this study was to determine whether chest DECT can be performed at reduced radiation doses lower than the standard-of-care dose, with an emphasis on images generated with post-processing techniques.

## MATERIALS AND METHODS

### Phantom experiment

A phantom study was performed to assess the reliability of CT numbers and image noise (defined as the standard deviation of the voxel values) on DECT images acquired at a reduced radiation dose. An anthropomorphic chest phantom (ATOM 701-B; CIRS Inc., Norfolk, VA, USA) was used for this experiment. Two plastic test tubes containing diluted contrast medium (iopamidol 370 mg/mL% diluted with saline at 1:20 and 1:40) were taped on the surface of the phantom. The phantom was scanned twice, with the standard-of-care and low-dose DECT protocols. All other scan parameters were kept constant, including scan length and scanned region.

**Figure 1:**
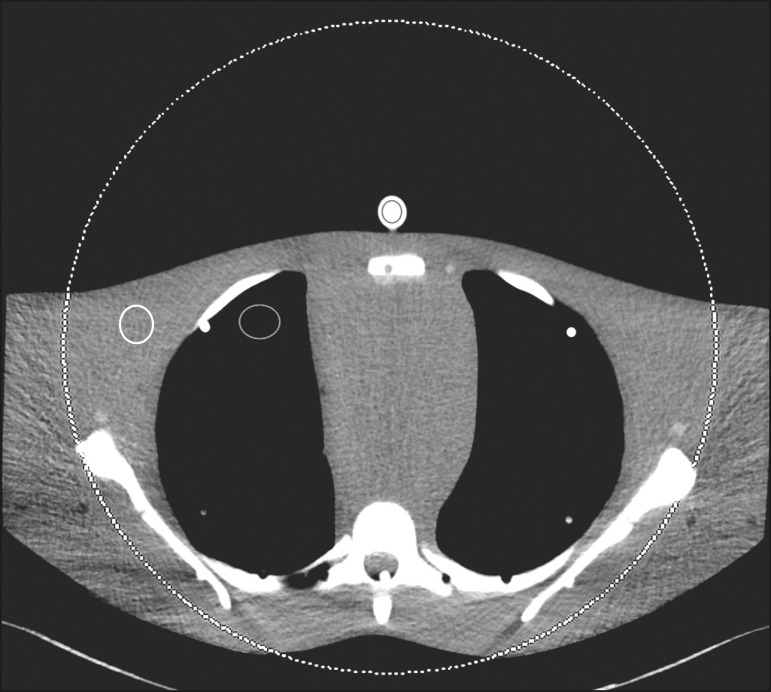
Transverse CT image of the anthropomorphic thoracic phantom. ROIs were drawn to measure HU at three different locations (soft tissues, right upper lung, and the test tubes filled with diluted contrast medium).

Circular regions of interest (ROIs) were drawn at 10 different sites in the chest wall and right upper lung area of the phantom to assess CT numbers (Hounsfield units [HU]) and their standard deviations (SDs) in both sets of images (Figure 1). CT numbers and SDs were also measured at two different locations in the test tubes filled with diluted contrast medium.

### Patient study

The Human Research Committee of our Institutional Review Board approved this prospective study, and all participating patients gave written informed consent. The study was conducted in accordance with the Health Insurance Portability and Accountability Act guidelines for research. Our institution received a research grant from Siemens Healthcare.

Two study co-investigators (AT and RC) reviewed the Radiology Information System to identify patients scheduled for a contrast-enhanced routine chest CT. Patients were considered eligible to participate in the study if they were well oriented, hemodynamically stable, and ≥ 56 years of age. Patients with cognitive impairment or other conditions that would make them unable to give informed consent for CT scanning were excluded, as were those undergoing emergency CT, those who were hemodynamically instable, those who were unable to hold their breath for at least 10 s, those with a known history of allergic reaction to contrast media, and those with a body mass index (BMI) above 32 kg/m^2^. Patients with a known history of interstitial lung disease were also excluded, because they had already been submitted to two CT acquisitions (one in the prone position and one in the supine position) at our institution. A total of 45 patients were invited to participate in the study. Of those, 19 declined and 26 gave written informed consent. Five patients subsequently withdrew from the study because they underwent chest CT in a single-energy CT scanner. Therefore, the final sample comprised 21 adult patients (8 men and 13 women). The mean age was 72 ± 7 years (range, 56-87 years) overall, 70 ± 5 years for the men, and 73 ± 8 years for the women. Clinical indications for CT included lung cancer staging and treatment response evaluation (n = 5); unresolved pneumonia (n = 2); and staging of extrathoracic malignancies (n = 14).

### Scanning techniques

All clinically indicated contrast-enhanced chest CT examinations included in our study were performed in a 64-row, dual-source, multidetector CT scanner (Somatom Definition Flash; Siemens Healthcare, Forchheim, Germany) with a z-flying focal spot (double z-sampling). All CT examinations were performed after intravenous administration of 65 mL of iodinated contrast medium (Isovue-370; Bracco Diagnostics, Princeton, NJ, USA). The contrast medium was injected at a rate of 2.5 mL/s, with a fixed delay (35 s) as a trigger to scan the patient. The scan parameters are summarized in Table 1. Each scan series had an identical duration (approximately 3 s).

**Table 1:** Scan parameters for standard-of-care and reduced-dose DECT.

Parameter	Standard-of-care DECT	Reduced-dose DECT
Voltage tube A (kV)	80	80
Voltage tube B (kV)	140 (with tin filter)	140 (with tin filter)
Quality reference (mAs)	180	90
Acquisition (mm)	128 × 0.6	128 × 0.6
Rotation time (s)	0.5	0.5
Pitch	1.2	1.2
Direction	Craniocaudal	Craniocaudal
Slice thickness (mm)	3	3
Slice increment (mm)	2	2
Kernel	I30f, strength 3	I30f, strength 3
Window	Mediastinum	Mediastinum

After an initial planning CT of the chest had been acquired, identical scan coverage (from the lung apices to the upper pole of the kidneys) was specified for the standard-of-care and reduced-dose image series. The reduced-dose image series was acquired within 10 s after the standard-of-care CT image series. For the reduced-dose protocol, the quality reference mAs (Care Dose 4D; Siemens Healthcare) was reduced in order to achieve a radiation dose that was approximately half of that prescribed in the standard-of-care protocol^(^^10^^)^. All others scanning parameters were kept constant between the two scan series. No additional intravenous contrast media was used for the reduced-dose image series. The volumetric CT dose index (CTDIvol) and dose-length product (DLP) were recorded for each image series. We also recorded the water-equivalent diameter and size-specific dose estimate (SSDE) for each patient, using radiation dose tracking software (Radimetrics Enterprise Platform; Bayer Inc., Whippany, NJ, USA), as previously described^(^^11^^)^. For all chest CT examinations, effective doses (EDs) were calculated by multiplying the DLP by a conversion coefficient of 0.014^(^^12^^)^.

### Image reconstruction

All standard-of-care and reduced-dose images were reconstructed with a vendor-specific iterative reconstruction technique known as sinogram-affirmed iterative reconstruction (SAFIRE; Siemens Healthcare), at S3 settings, with the standard-of-care, medium-smooth soft-tissue reconstruction kernel (I30f). Because we did not include patients with interstitial lung disease (which would require sharp kernels to provide better spatial resolution and edge detection), we chose to reformat all images using the soft-tissue kernel (which provides optimal image contrast, at the cost of spatial resolution).

Blended images (80/Sn140 kV) were reconstructed in the transverse orientation at a slice thickness of 3 mm and an increment of 2 mm. These images were uploaded to a dedicated workstation with DECT image processing software (Syngo.via; Siemens Healthcare), in order to generate virtual monochromatic images at 40 keV and 60 keV, perfused blood volume images, and virtual non-contrast-enhanced images. Low-energy virtual monochromatic images were chosen for the comparison because they have similar or less noise than do the blended images and can enhance the conspicuity of iodine^(^^13^^-^^16^^)^. The 40 keV images were selected because they are closest to the K-edge of iodine (33 keV). The 60 keV images were selected because they have less noise than do 40-50 keV images but have contrast superior to that previously reported for 65-70 keV images in the evaluation of the mediastinal and pulmonary vessels^(^^8^^,^^17^^)^. All scan parameters and patient information were anonymized prior to the subjective evaluation of images (by AT and RC).

### Subjective assessment

Chest CT images were assessed independently by two board-certified experienced thoracic radiologists (EF and SD, with 10 and 15 years experience, respectively) on a DICOM-compliant image viewer (ClearCanvas Workstation; ClearCanvas Inc., Toronto, Canada). Both radiologists were blinded to the dose employed in each image series. Each radiologist performed a side-by-side comparison of anonymized standard-of-care and reduced-dose images. Because this was a proof-of-concept study designed to determine whether the image quality was comparable between the two protocols and whether the increase in noise could compromise the assessment of a lesion, the side-by-side approach was deemed appropriate.

Each radiologist assessed mediastinal and lung lesions as well as normal anatomic structures (such as lung fissures, the sub-segmental bronchial wall, the pericardium, and sub-centimeter mediastinal lymph nodes) on virtual monochromatic images at 60 keV, using a two-point scale (1 = suboptimal visualization; and 2 = optimal visualization) for overall image quality. Image quality characteristics assessed in this study have been described previously^(^^18^^)^.

Two different radiologists (SM and AO, with 10 and 8 years of experience, respectively) independently assessed the effect of reduced-dose DECT images on diagnostic information. Each radiologist was blinded to the identity of each image series and was asked to identify lesions in the lung parenchyma and mediastinum on two different sets of images. The first set included virtual monochromatic images at 40 keV (reduced-dose protocol images) and the second set included virtual monochromatic images at 60 keV (standard-of-care protocol images). For each dose level, diagnostic confidence was graded on a five-point Likert scale^(^^19^^)^: 5 = abnormal structures clearly visible with good demarcation (excellent); 4 = abnormal structures visible with blurring but without restriction of diagnosis (good); 3 = abnormal structures visible, with blurring and uncertainties about the evaluation (poor); 2 = abnormal structures barely visible with unreliable interpretation (unacceptable); and 1 = abnormal structures not seen (none). The 40 keV monochromatic images were used for reduced-dose DECT in order to maximize contrast enhancement in an acquisition that was slightly delayed in comparison with the initially acquired standard-of-care images.

### Objective assessment

Image noise and signal data (CT numbers in HU) were obtained by drawing three ROIs: in the tracheal lumen just above the carina, in the mid-thoracic vertebral body, and in paraspinal muscle at the same level. CT numbers were also measured in the right pulmonary artery. Circular ROIs (0.5-0.8 cm^2^ in area) were drawn by a single investigator (RC) on virtual monochromatic images at 60 keV and 40 keV from the standard-of-care and reduced-dose datasets.

The contrast-to-noise ratio (CNR) and signal-to-noise ratio (SNR) were also calculated^(^^20^^)^. The ROI in the trachea was used as a reference to calculate the CNR. Given the 10-s delay between the standard-of-care and reduced-dose image series, quantitative measurements of iodine concentration were not performed in the perfused blood volume images.

### Statistical analysis

The data were analyzed using the SPSS Statistics for Macintosh, version 23.0 (IBM Corp., Armonk, NY, USA). Student's t-test was used in order to compare image noise and CT numbers in the right pulmonary artery between the two groups. The Wilcoxon signed-rank test was used in order to assess differences in subjective image quality characteristics between standard-of-care and reduced-dose DECT. Interobserver agreement between the two radiologists was assessed with Cohen's kappa statistic. Agreement was regarded as poor at a kappa ≤ 0.20, fair at a kappa of 0.21-0.40, moderate at a kappa of 0.41-0.60, good at a kappa of 0.61-0.80, and excellent at a kappa > 0.80. One-way analysis of variance was used in order to compare mean HU values on the phantom study between the standard-of-care and reduced-dose groups. Values of *p* < 0.05 were considered statistically significant.

## RESULTS

As can be seen in Table 2, the phantom study revealed no significant differences between standard-of-care and reduced-dose DECT images in terms of the mean CT numbers in soft tissues (*p* = 0.515) and lung parenchyma (*p* = 0.888). We also found no significant difference in image noise between the standard-of-care and reduced-dose protocols (*p* = 0.406). The demographics of the patient sample are summarized in Table 3.

**Table 2:** Mean CT numbers in the anthropomorphic phantom experiment.

	Standard-of-care DECT	Reduced-dose DECT
Location	(HU ± SD*)	(HU ± SD*)
Soft tissues (60 keV)	31.5 ± 14.5	32.2 ± 14.0†
Soft tissues (40 keV)	32.5 ± 26.0	30.3 ± 28.2†
Lung parenchyma (60 keV)	-792.9 ± 10.9	-791.3 ± 12.5†
Lung parenchyma (40 keV)	-780.3 ± 18.0	-777.9 ± 21.4†
ICM, 1:20 dilution (40 keV)	1495.2 ± 49.1	1487.7 ± 53.2†
ICM, 1:20 dilution (60 keV)	673.3 ± 20.8	667.1 ± 23.5†
ICM, 1:40 dilution (40 keV)	780.8 ± 35.8	767.5 ± 34.4†
ICM, 1:40 dilution (60 keV)	346.1 ± 13.4	339.4 ± 19.2†

ICM, iodinated contrast media.

* The SD of the HU represents objective image noise.

† No significant difference between standard-of-care and reduced-dose DECT.

**Table 3:** Characteristics of patients included in our study.

Characteristics	Male patients	Female patients
Number of patients	8	13
Age, in years (mean + SD)	70 ± 5	73 ± 8
Weight (kg)	81 ± 11	65 ± 11
BMI, in kg/m^2^ (mean + SD)	25 ± 3	24 ± 3
BMI (n)		
≤ 20 kg/m^2^	0	1
20.1-25 kg/m^2^	4	7
25.1-30 kg/m^2^	3	5
30.1-32 kg/m^2^	1	0

### Subjective assessment

#### Side-by-side comparison on monochromatic images at 60 keV

**Figure 2:**
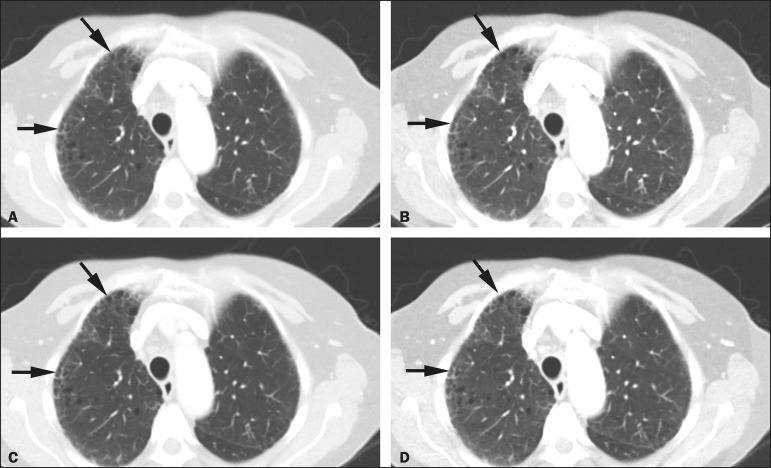
Transverse contrast-enhanced DECT images of a 73 year-old-female (BMI = 23.4 kg/m^2^) who was referred for staging of lung cancer. Standard-of-care monochromatic images at 60 keV (**A**) and 40 keV (**B**) demonstrate findings consistent with interstitial lung disease (pulmonary fibrosis) in the right upper lobe. Reduced-dose monochromatic images at 60 keV (**C**) and 40 keV (**D**) demonstrating identical findings. Overall image quality was deemed optimal at both dose levels.

**Figure 3:**
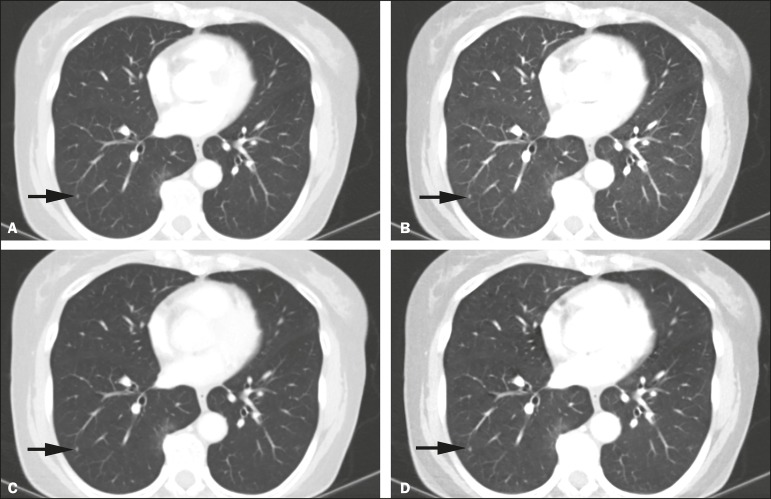
Contrast-enhanced DECT to investigate persistent cough in a 70 year-old-male (BMI = 25.9 kg/m^2^). Standard-of-care monochromatic images at 60 keV (**A**) and 40 keV (**B**) showing an indeterminate small nodule (arrow) in the right lower lobe. Monochromatic images at 60 keV (**C**) and 40 keV (**D**) from reduceddose DECT showing the same nodule. Overall image quality was considered optimal at both dose levels.

No differences were observed between the standard-of-care and reduced-dose DECT images in terms of the visualization of normal pulmonary structures (lung fissures and the sub-segmental bronchi wall), sub-centimeter lymph nodes (in the paratracheal, subcarinal, and hilar chains), and the pericardium. All 39 of the mediastinal and parenchymal lesions seen on virtual monochromatic images in the standard-of-care DECT were also seen in the reduced-dose DECT, as depicted in Figures 2 and 3.

Abnormalities visualized on the DECT images included sub-centimeter pulmonary noncalcified solid nodules (n = 17); pulmonary noncalcified solid nodules > 1 cm (n = 1); emphysema (n = 7); postoperative changes (n = 5); mediastinal and hilar lymphadenopathy (n = 3); a mosaic attenuation pattern (n = 1); tree-in-bud nodules (n = 2); lung masses (n = 1); bone metastasis (n = 1); pulmonary fibrosis (n = 1); and sternal osteomyelitis (n = 1). Overall subjective image quality was deemed optimal on 60 keV monochromatic images for the reduced-dose DECT protocol in all 21 cases, with perfect agreement between the two readers (kappa = 1).

#### Lesion detection performance on monochromatic images at 40 keV

The number and type of mediastinal lesions detected on the 40 keV (reduced-dose) images were also seen on the 60 keV (standard-of-care) images by the assigned readers. The reduced-dose image series received excellent scores for diagnostic confidence, with perfect interobserver agreement (kappa = 1).

Regarding the pulmonary parenchyma findings, the reduced-dose protocol allowed the detection of pulmonary nodules as small as 2 mm. Good intraobserver agreement (kappa = 0.72) and good interobserver agreement (kappa = 0.65) were observed. When noncalcified pulmonary nodules less than 5 mm were excluded from the analysis, the interobserver agreement increased substantially, from good to excellent (kappa = 0.85). Diagnostic confidence was rated as good (4 points) in 3 patients and as excellent (5 points) in 18 patients, the findings being considered diagnostic for clinical purposes in all cases.

### Objective assessment

Table 4 summarizes the results of the objective image quality evaluation. The reduced-dose image series showed less noise (down to 12%) in the tracheal lumen, although it showed an increase in noise (up to 44%) in muscle and bone tissues. Nevertheless, that was accompanied by an increase in the CNR (up to 20%).

**Table 4:** Objective assessment of image noise, SNR, and CNR for standard-of-care chest DECT and reduced-dose chest DECT.

	Standard-of-care DECT			Reduced-dose DECT			% Difference	
Variable	60 keV	40 keV		60 keV	40 keV		60 keV	40 keV
Image noise (mean ± SD)								
Trachea	8.1 ± 2.6	8.8 ± 3.3		7.1 ± 3.1	8.1 ± 5.8		-12%	-8%
Muscle	13.5 ± 3.0	23.6 ± 5.0		18.3 ± 3.4	34.0 ± 6.2		35%	44%
Bone	24.8 ± 8.1	39.8 ± 14.8		28.9 ± 6.2	48.6 ± 13.4		16%	22%
SNR (mean ± SD)								
Trachea	131.6 ± 7.2	125.1 ± 40.2		157.3 ± 51.3	151.5 ± 53.3		19%	21%
Muscle	3.9 ± 1.0	2.3 ± 0.9		3.1 ± 0.9	1.8 ± 0.8		-21%	-22%
Bone	8.1 ± 4.0	8.6 ± 4.0		6.6 ± 2.5	7.3 ± 3.2		-19%	-15%
CNR (mean ± SD)								
Muscle	124.8 ± 37	118.5 ± 38.6		148.6 ± 48.8	142.5 ± 51.1		19%	20%
Bone	108.1 ± 32	86.8 ± 27.3		128.2 ± 41.3	101.7 ± 35.7		19%	17%

On the 60 keV virtual monochromatic images, the mean attenuation in the right pulmonary artery was 227 ± 73 HU for reduced-dose images and 410 ± 122 HU for standard-of-care images. As expected, the 40 keV virtual monochromatic images yielded attenuation values that were higher (up to 129%) than those observed for the 60 keV virtual monochromatic images. It is worth noting that the mean attenuation in the right pulmonary artery was found to be slightly higher on 40 keV images acquired with the reduced-dose protocol (453 ± 103 HU) than on 60 keV images acquired with the standard-of-care protocol (404 ± 119 HU), despite the 10-s delay between the protocols.

### Radiation dose

The respective mean CTDIvol, SSDE, DLP, and ED values for standard-of-care and reduced-dose DECT, respectively, were as follows: 6.0 ± 1.3 and 3.0 ± 0.6 mGy (*p* < 0.001); 7.0 ± 1.2 and 4.0 ± 0.6 mGy (*p* < 0.001); 194 ± 60 and 107 ± 30 mGy.cm (*p* < 0.001); and 2.7 ± 0.8 and 1.5 ± 0.4 mSv (*p* < 0.001). To our knowledge, those are the lowest CT dose metrics ever reported in a study of patients undergoing DECT of the chest. In our study, the overall mean per-patient ED (including the standard-of-care and reduced-dose image series) was 4.2 ± 1.2 mSv, which is lower than the 7 mSv reported for chest CT in the literature^(^^21^^)^.

## DISCUSSION

Our results demonstrate that, in patients with a BMI < 32 kg/m^2^, DECT of the chest can be performed at reduced doses (down to a CTDIvol of 3.0 ± 0.7 mGy and a DLP of 107 ± 30 mGy.cm) without a loss of diagnostic information. The reduction in the radiation dose did not compromise the visibility of subtle thoracic anatomic structures such as small nodules, lung fissures, bronchial walls, and subsegmental pulmonary vessels on the processed images. We also demonstrated that virtual monochromatic images at 40 keV and 60 keV, generated from the reduced-dose images, can still be used as reliable diagnostic tools to evaluate mediastinal and pulmonary lesions.

Our findings run contrary to those of the initial studies regarding DECT protocols, which showed that the technique exposed patients to high doses of radiation^(^^22^^,^^23^^)^. In addition, recent publications have reported the use of low-radiation-dose protocols and the possibility of eliminating one or more scanning phases for certain clinical indications, such as those prompting CT urography or CT angiography^(^^24^^,^^25^^)^. In this regard, the radiation dose reduction achieved by DECT scanners can be even more impressive.

The radiation dose employed for DECT in our study is lower than those reported in previous studies^(^^8^^)^. In a phantom study, Schenzle et al.^(^^5^^)^ reported that chest DECT can be performed at a CTDIvol of 5.4 mGy with an ED of 2.6 mSv. Those authors also documented a better CNR with DECT than with single-energy CT at 120 kV. De Broucker et al.^(^^23^^)^ reported a mean DLP of 403.4 mGy.cm for a CT angiography examination using a dual-source DECT scanner. Hwang et al.^(^^26^^)^ reported an ED of 1.78 mSv for a reduced-dose chest CT study, using a dual-source DECT scanner with a single-energy acquisition (120 kV).

Our study also demonstrated the benefit of lower energy virtual monochromatic images (40 keV vs. 60 keV) in the evaluation of the pulmonary artery. In most of our patients, the attenuation in the pulmonary artery was better on 40 keV (reduced-dose protocol) images than on 60 keV (standard-of-care protocol) images, even when the 10-s delay was taken into consideration. That is explained by the fact that the K-edge of iodine (approximately 33 keV) is closer to 40 keV than to 60 keV. This ability of DECT to improve contrast enhancement can allow the volume of contrast medium injected for CT scanning to be reduced^(^^27^^)^, which could be especially beneficial for patients at risk for contrast-induced nephropathy.

Our study has several limitations. The additional radiation dose, incurred through the acquisition of reduced-dose DECT images in patients who also underwent standard-of-care DECT, although low, limited our study sample size. However, we mitigated the potential risks of the additional radiation dose by including only patients who were over 56 years of age. In addition, our study involved the use of only one DECT scanner from a single vendor, because it was not possible to reduce the radiation dose by 50% (in relation to the standard-of-care dose) on our other DECT scanners with rapid kV-switching technique. The rapid kV-switching technique only works at presets of fixed doses and tube current, and our standard-of-care DECT chest protocol uses preset with the lowest allowed dose. Furthermore, we used a single iterative reconstruction setting (SAFIRE S3), although it was found to be sufficient in all patients and is routinely used in our clinical practice. Moreover, we did not include patients with a BMI above 32 kg/m^2^ or patients with interstitial lung disease, and it is therefore unknown how reduced-dose DECT of the chest will perform in such cases. Further studies are needed in order to assess the reliability of quantitative measurements of iodine on material decomposition images from reduced-dose DECT of the chest and other body regions. Finally, as per our standard-of-care clinical practice, we only used one kernel (I30f) for the evaluation of all image series for the lungs and the mediastinum. Therefore, our study did not address the effect of reduced-dose DECT on the interpretation of lung findings with high spatial frequency or sharper kernels.

In summary, chest DECT can be performed at a substantially reduced dose (down to 3.0 mGy) in patients with a BMI below 32 kg/m^2^. The reduced-dose monochromatic images at 40 keV and 60 keV can be used in evaluating normal and abnormal findings in the thorax without a loss of diagnostic information.
